# A Case of CRP-Negative Giant Cell Arteritis Detected by Contrast-Enhanced Orbital MRI

**DOI:** 10.7759/cureus.98537

**Published:** 2025-12-05

**Authors:** Midori Nakazawa, Sotaro Mori, Yukina Tanimoto, Feibi Zeng, Jun Saegusa, Makoto Nakamura

**Affiliations:** 1 Division of Ophthalmology, Department of Surgery, Kobe University Graduate School of Medicine, Kobe, JPN; 2 Department of Rheumatology and Clinical Immunology, Kobe University Graduate School of Medicine, Kobe, JPN; 3 Department of Diagnostic and Interventional Radiology, Kobe University Graduate School of Medicine, Kobe, JPN

**Keywords:** arteritic anterior ischemic optic neuropathy, c –reactive protein (crp), giant cell arteritis (gca), optic disc swelling, s: magnetic resonance imaging

## Abstract

Giant cell arteritis (GCA) is a vision-threatening emergency that can cause irreversible visual loss due to arteritic ischemic optic neuropathy. We report a case in which C-reactive protein (CRP) blood tests and whole-body imaging showed no signs of inflammation, but an abnormal signal in the superficial temporal artery on MRI led to the diagnosis.

A 90-year-old man presented with a two-week history of decreased visual acuity in his left eye. At the initial visit, his visual acuity was 20/25 in the right eye and no light perception in the left eye. Bilateral optic disc swelling and right-sided temporal pain were noted. Laboratory tests showed negative CRP and only mildly elevated erythrocyte sedimentation rate, while whole-body CT and temporal artery ultrasound demonstrated no inflammatory changes. Although GCA does not apply to the internal medicine department, emergency steroid pulse therapy was started to preserve visual function. During treatment, orbital MRI performed to differentiate from optic neuritis revealed contrast enhancement in the right superficial temporal artery, corresponding to the site of headache. Subsequent temporal artery biopsy demonstrated medial rupture, confirming the diagnosis of GCA.

In cases where GCA is clinically suspected and visual impairment is present, prompt initiation of treatment is essential even in the absence of clear inflammatory findings, and orbital contrast-enhanced MRI may provide critical diagnostic clues.

## Introduction

Giant cell arteritis (GCA) is an ophthalmologic emergency that can cause irreversible visual loss due to arteritic anterior ischemic optic neuropathy (A-AION) [1]. The diagnosis is straightforward when typical clinical features are present. However, in atypical cases, hesitation to initiate treatment due to diagnostic uncertainty may result in permanent visual impairment, severely affecting the patient’s vision and quality of life.

We report a case of GCA in which initial blood tests showed no elevation in C-reactive protein (CRP) or erythrocyte sedimentation rate (ESR), and neither CT nor ultrasound revealed any inflammatory findings. At the time of presentation, the patient did not meet the 2022 ACR/EULAR classification criteria for GCA, jointly developed by the American College of Rheumatology (ACR) and the European League Against Rheumatism (EULAR) [1]. However, contrast-enhanced magnetic resonance imaging (MRI), performed to investigate differential diagnoses, revealed high signal intensity with contrast enhancement in the superficial temporal artery, suggestive of inflammation, ultimately leading to the diagnosis of GCA.

Although classification criteria are useful, they are not definitive for diagnosis. In atypical cases, especially when urgent treatment is necessary to prevent irreversible visual loss, clinicians must proceed with simultaneous diagnostic evaluation and therapeutic intervention. We report this case to highlight these considerations.

## Case presentation

A 90-year-old man with no systematic medical history, except for previous bilateral pterygium surgery, was referred to our hospital by a local ophthalmologist with complaints of decreased vision in his left eye for two weeks. At the initial examination, best-corrected visual acuity was 20/25 in the right eye and no light perception in the left eye. A relative afferent pupillary defect (RAPD) was present in the left eye. Ophthalmological examination revealed moderate cataracts and bilateral optic disc swelling.

**Figure 1:**
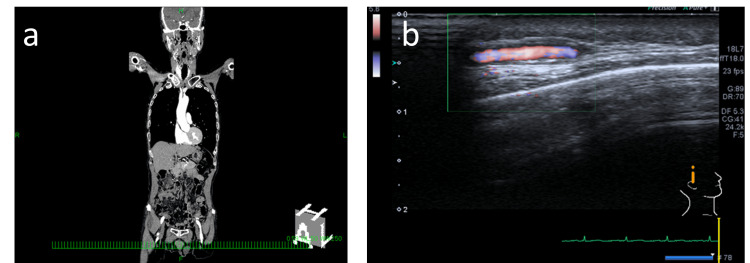
Images of (a) Whole-body contrast-enhanced computed tomography, and (b) Temporal artery ultrasonography These findings did not suggest vasculitis.

Given the acute visual loss, optic disc swelling, and accompanying right-sided temporal head pain, GCA was suspected. However, laboratory testing showed a normal CRP level and only a mildly elevated ESR of 24 mm/h (Table 1). Whole-body contrast-enhanced computed tomography (CT; Figure 1a) and temporal artery ultrasonography (Figure 1b) revealed no apparent signs of inflammation. At this stage, the patient did not meet the 2022 ACR/EULAR classification criteria for GCA [1].

**Table 1:** Blood test results before treatment

Complete blood count	Values	Reference range
White Blood Cell (/µl)	8,000	3,300-8,600
Red Blood Cell (×10^6^/μl)	4.11	3.86-4.92
Hemoglobin (g/dl)	12.4	11.6-14.8
Platelet (×10^3^/μl)	169	158-348
Biochemistry		
Na (mmol/l)	139	138-145
K (mmol/l)	4.7	3.6~4.8
Cl (mmol/l)	103	101~108
Blood Urea Nitrogen (mg/dl)	19.9	8.0-20.0
Creatinine (mg/dl)	0.87	0.46-0.79
Erythrocyte Sedimentation Rate (mm/h)	24	<0.14
C-Reactive Protein (mg/dl)	0.04	<15
Tumor-associated test		
Soluble interleukin 2 receptor (U/ml)	577	156.6-474.5

**Figure 2:**
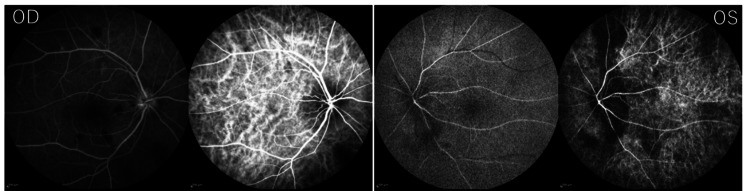
Fundus fluorescein (left side) and indocyanine green angiography (right side) images Fluorescein angiography and indocyanine green angiography revealed filling defects around the optic disc in both eyes.

Nevertheless, due to the possibility of GCA and the urgent need to preserve visual function, the patient was admitted the same day and started on high-dose intravenous methylprednisolone (1 g/day for three days). Fundus fluorescein and indocyanine green angiography performed the following day revealed delayed choroidal perfusion around the optic discs in both eyes, consistent with bilateral ischemic optic neuropathy (Figure 2).

**Figure 3:**
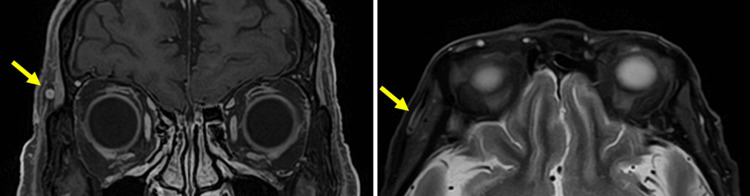
Orbital contrasted MRI images before treatment Left, T1 gadolinium-enhancing image: Right, STIR (Short Tau Inversion Recovery) image, Contrast enhancement was observed in the right superficial temporal artery.

**Figure 4:**
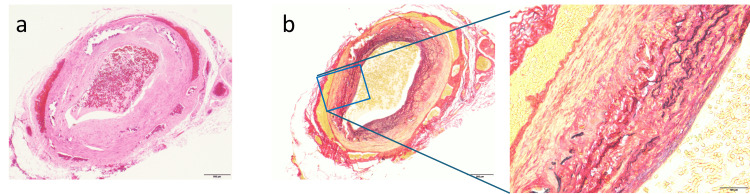
Microphotograph of temporal artery biopsy The sections were stained with (a) hematoxylin-eosin, and (b) Elastic Van Gieson. The scale bar indicates 500 µm in low-magnification images and 100µm in high-magnification images.

To differentiate the condition from optic neuritis, a contrast-enhanced orbital MRI was performed on day three of treatment, revealing high signal intensity of the arterial wall on the short tau inversion recovery (STIR) image and contrast effect on the post-contrast T1-weighted image, consistent with arterial mural inflammation (Figure 3). Although the rheumatology and clinical Immunology department initially ruled out GCA and advised against a temporal artery biopsy, the MRI findings prompted a biopsy, which was performed on day 10 of treatment. The initial pathological diagnosis was negative for active inflammation or multinucleated giant cells, but subsequent staining with Elastic Van Gieson (EVG) revealed a tear in the tunica media (Figure 4), supporting a diagnosis of GCA.

The patient’s severe right-sided temporal head pain began to improve by day five of treatment and resolved completely by day 15. Despite treatment, the pupillary light reflex was lost bilaterally by day four, and light perception was absent in both eyes. Following pulse therapy, the patient was transitioned to oral prednisone at 50 mg/day for 20 days, which was tapered by 10 mg/week down to 20 mg/day, followed by 5 mg reductions every two weeks.

By day 22, visual acuity in the right eye had improved to 20/200. At the final follow-up (five months after treatment), the right eye retained some peripheral visual field, while the left eye had no light perception.

## Discussion

This case represents a rare presentation of GCA in which no inflammatory findings were detected on blood tests, CT, or ultrasound imaging. Fundus angiography revealed findings suggestive of simultaneous ischemic optic neuropathy in both eyes, and the patient’s medical history included no conditions that could lead to non-arteritic AION (NA-AION). Therefore, the clinical course was considered consistent with bilateral A-AION due to GCA.

In typical cases of GCA, blood tests reveal elevated CRP and ESR. However, Kermani et al. reported that among 177 biopsy-proven GCA patients, 7 (4%) had normal ESR and CRP levels [2]. Similarly, Parikh et al. found that in 119 biopsy-confirmed GCA patients, the sensitivity of ESR was 76-86%, while that of CRP was 97.5%; only one patient (0.8%) had normal values for both markers [3]. While these inflammatory markers are highly sensitive and clinically useful, caution is warranted, as multiple case reports have described GCA patients with normal CRP and ESR levels [4-9].

In our case, whole-body contrast-enhanced CT also showed no evidence of vascular inflammation. According to a Japanese nationwide study, the sensitivity of contrast-enhanced CT for GCA is approximately 82.0% [10]. International studies have reported sensitivities ranging from 73.3% to 95%, with 18F-fluorodeoxyglucose PET/CT demonstrating higher sensitivity [11]. However, in cases presenting with A-AION, such as this case, the urgency of diagnosis often precludes early use of PET/CT. Moreover, the reported sensitivity of temporal artery ultrasound varies widely, ranging from 54% to 89% depending on patient background and operator experience [12-14].

According to the 2022 ACR/EULAR classification criteria [1], this patient met the clinical domains of “sudden visual loss” and “new-onset temporal headache.” However, under the domain of “abnormal examination of the temporal artery,” the initial medical record documented no diminished pulse, tenderness, or cord-like thickening. No additional clinical criteria were satisfied. Although ESR was mildly elevated at 24 mm/h, it did not meet the threshold of ≥50 mm/h required by the classification criteria.

Regarding temporal artery biopsy, while the classification criteria state that there are no strictly defined histopathological requirements, supportive features include giant cell infiltration, mononuclear leukocyte infiltration, and fragmentation of the internal elastic lamina. In this case, a superficial temporal artery biopsy was performed on day 10 of steroid treatment. The initial histopathological assessment showed none of these typical findings. Although steroid pulse therapy can reduce vascular inflammation rapidly, classic histologic features often persist for several days to two weeks following treatment initiation [15]. Importantly, due to the risk of irreversible vision loss, ACR guidelines emphasize that treatment should not be delayed for biopsy confirmation when clinical suspicion is high [1].

In this case, additional EVG staining revealed disruption of the tunica media. EVG staining highlights elastic fibers and is particularly useful in identifying structural changes in vessel walls, such as fragmentation of the internal elastic lamina and medial disruption. While these changes are not specific to GCA and may also occur with age-related vascular degeneration or atherosclerosis [16], the absence of other identifiable causes of the temporal headache led us to consider GCA the most likely diagnosis.

However, in this case, fundus angiography revealed choroidal filling defects around the optic disc in both eyes, strongly suggesting GCA. Such defects are indicative of ischemic optic neuropathy. The bilateral involvement and absence of significant systemic risk factors-such as renal dialysis-support a diagnosis of A-AION.

Differential diagnoses for sudden vision loss of this patient included optic neuritis due to systemic inflammatory diseases such as syphilis or sarcoidosis; infiltrative optic neuropathies caused by neoplastic, granulomatous, or infectious infiltration; compressive optic neuropathy from orbital or perioptic lesions; diabetic papillopathy; and toxic, metabolic, or hereditary optic neuropathies. Although optic neuritis was still considered, despite the patient’s advanced age, orbital contrast-enhanced MRI was performed to aid in differentiation.

In GCA, contrast-enhanced MRI typically shows mural inflammation of the superficial temporal artery. These findings are best visualized on T1-weighted gadolinium-enhanced sequences, demonstrating mural thickening and enhancement [17]. This imaging pattern has been reported to have a sensitivity of 75% and specificity of 89% in patients with GCA [18]. Although the primary aim of imaging in this case was to rule out optic neuritis, these highly specific features of GCA were incidentally revealed.

From an ophthalmologic perspective, patients with GCA frequently present with sudden visual loss, which can mimic optic neuritis. Therefore, it is essential to assess enhancement of the superficial temporal artery on contrast-enhanced orbital MRI not only in patients suspected of GCA, but also in those with presumed optic neuritis.

## Conclusions

This case illustrates the diagnosis of GCA based on fluorescein angiography and contrast-enhanced orbital MRI findings. Unfortunately, both choroidal filling defects on fundus angiography and superficial temporal artery enhancement on MRI features with high specificity for GCA are not included in the 2022 ACR/EULAR classification criteria. This likely reflects the fact that the criteria were developed primarily from an internist’s perspective, rather than an ophthalmologist’s. Furthermore, even when fundus angiography clearly suggests GCA, internists may have limited familiarity with interpreting these findings and may not fully appreciate their diagnostic weight.

While classification criteria provide useful guidance, they are not definitive. In atypical cases such as this, where early treatment is essential to prevent irreversible visual loss, it is critical to consider the full clinical picture, weigh alternative diagnoses carefully, and pursue prompt diagnosis and treatment when the suspected disease, such as GCA-requires urgent intervention.
